# Gout is not associated with the risk of fracture: a meta-analysis

**DOI:** 10.1186/s13018-019-1317-4

**Published:** 2019-08-27

**Authors:** Fanxiao Liu, Jinlei Dong, Dongsheng Zhou, Qinglin Kang, Fei Xiong

**Affiliations:** 1 Department of Orthopaedics, Shandong Provincial Hospital Affiliated to Shandong University, No.324, Road Jing Wu Wei Qi, Jinan, 250021 Shandong China; 2 Department of Orthopedic Surgery, Shanghai Jiao Tong University Affiliated Sixth People’s Hospital, Yishan Road 600, Shanghai, 200233 China

**Keywords:** Gout, Meta-analysis, Fracture risk, Urate-lowering drugs

## Abstract

**Background:**

Numerous quantitatively based studies measuring the association between gout and the risk of fractures remain inconclusive. In order to determine whether gout could increase the risk of fractures, a meta-analysis was performed systematically.

**Methods:**

Electronic databases, MEDLINE/PubMed, Embase, and Cochrane Library were systematically searched to identify studies evaluating the association of gout and the risk of fractures. No restrictions on language, publication date, or journal of publication were imposed. Meta-analysis was performed to pool the outcome estimates of interest such as fracture incidence, fracture risk, and fracture risk in different sites and at different time points in the follow-up period.

**Results:**

Screening determined that seven studies involving a total of 684,964 participants (151,002 in the gout group and 533,962 in the control group) were deemed viable for inclusion in the meta-analysis. The results of the analysis showed that gout would not significantly have a relatively higher risk of any fracture (RR = 1.11, 95% CI 0.98–1.26). Subgroup analysis showed consistent results for sexuality (female: RR = 1.13, 95% CI 0.93–1.37; male: RR = 0.99, 95% CI 0.91–1.07) and several occurring sites (humerus, wrist, vertebra, hip, upper limbs, and lower limbs). Additionally, the results demonstrated that urate-lowering drugs prescribed early during disease had neither adverse nor beneficial effect on the long-term risk of fractures (RR = 0.89, 95% CI 0.76–1.05).

**Conclusions:**

This meta-analysis confirmed that gout was not associated with an increased risk of fractures. Urate-lowering drugs prescribed early during the course of disease had neither adverse nor beneficial effect on the long-term risk of fractures.

**Electronic supplementary material:**

The online version of this article (10.1186/s13018-019-1317-4) contains supplementary material, which is available to authorized users.

## Introduction

Gout affected 2.4% of adults in the UK [1] and more than eight million Americans [2], which was one of the most common rheumatic diseases [3]. The growing recognition indicated that gout did not just occur in men, but also in women, and caused a substantial disease burden with the significantly higher cost, morbidities, and mortality [4–8]. Additionally, hyperuricemia, a characteristic in gout, was reported to be an independent risk factor for cardiovascular diseases, diabetes, and even death [9–12]. In recent decades, concern has mounted regarding the association between gout and bone health. It was generally believed that chronic inflammation had a negative effect on the bones due to the stimulation of the inflammatory cascade and production of pro-inflammatory cytokines, so gout, a type of inflammatory arthritis, has been hypothesized to be associated with a high risk of fractures [13, 14]. However, the increased serum level of uric acid (UA) in gout [15], which is the degradation production of purine compounds [16], made the relationship inconsistent. UA may affect bone health, including resorption and formation, through its two-way control of oxidative stress due to its antioxidant or pro-oxidant properties [17], and several prior studies on the association between hyperuricemia and bone mineral density (BMD) also demonstrated conflicting results [18–22]. Meanwhile, the urate-lowering therapy faced a dilemma when considering the relationship with fracture. Therefore, the indistinct interaction of these factors made it challenging to determine whether gout was protective or detrimental to bone health.

Numerous quantitatively based studies debating the association between gout and the risk of fractures remain inconclusive. Tzeng et al. [23] reported that gout history increased the fracture risk, which was consistent with the results of a Chinese adult-based research conducted by Wang et al. [24]. However, the relationship became variable when focus on different fracture sites in the study performed by Paik et al. [17], in which the risk of fractures raised in the hip but not in the wrist. Kim et al. [25] conducted a large-scale epidemiological study and demonstrated the neutral association between gout and bone health, and Sultan et al. [1] reported the similar results in a nationally representative cohort research. Additionally, a population-based study in Taiwan demonstrated a lower fracture risk among gout patients prescribed urate-lowering therapy [23], while a 9% higher risk was found in a Denmark registry-based study [26]. Hence, it was difficult to clarify the associations of gout and its related treatment with the risk of fractures based on these published observational studies.

The aim of this study was to assess precisely the risk of fractures among patients with gout and estimate the potential effect of urate-lowering therapy on the risk. Furthermore, several subgroup analyses based on the gender, fracture sites, and etc. were performed to reveal more influencing factors.

## Materials and methods

The checklist of the Preferred Reporting Items for Systematic Reviews and Meta-Analyses (PRISMA) statement was followed in the conduction of this investigation (Additional file 1). Electronic searches, reference lists screening, study selection, data extraction, assessment of the risk of bias, and pooling of outcome estimates were performed by two investigators (LFX and XF) independently. Any disagreement was resolved by consensus or judgment of an arbitrator (DJL). Informed consent or ethics approval was not needed because all required data were retrieved from published articles.

### Data sources and search strategy

Three electronic databases including MEDLINE/PubMed, Embase, and Cochrane Library (from inception to Feb 1, 2019) were searched through an iterative process using a combination of keywords and mesh terms “gout,” “arthritis,” or “uric acid” AND “fracture,” “bone fracture,” or “broken bone.” No restrictions on language, date, or journal of publication were imposed. Additionally, reference lists of related literature (reviews, meta-analyses, and included studies) about gout patients and associated fracture risk were carefully screened to retrieve additional eligible studies not identified by electronic database searching.

### Study screening and selection

Studies were included in the qualitative analysis if they confirmed criteria listed as below: (1) participants, subjects with a primary diagnosis of gout; (2) control group, subjects with no gout; (3) incidence of fractures was assessed in the gout group and control group; and (4) randomized/quasi-randomized/cluster controlled clinical trials and retrospective/prospective cohort studies.

Exclusion criteria were (1) study protocols, letters, correspondence, and conference addresses; (2) experimental studies or animal studies; or (3) trials without sufficient data to obtain endpoint outcomes of interest.

### Data extraction

Following information was collected from included articles: first author’s family name, year of publication, study design, inclusion criteria of participants, source of data, number of participants in the gout group and the control group, demographic and clinical characteristics of participants (age, sex, and comorbidities), follow-up, evaluation endpoint outcomes of interest (any fracture, upper limb fracture, lower limb fracture), and the drugs of urate-lowering therapy. Risk ratios (RR) and its related 95% confidence intervals (CIs) were directly extracted from original articles if provided or calculated from individual patients’ data.

### Risk of bias

Newcastle-Ottawa Scale (NOS) [27] was used to assess the risk of bias for the quality of non-randomized studies, in which each study will be assessed according to eight items belonging to three categories: (1) study groups selection, (2) comparability of groups, and (3) outcome of interest. One score was earned if a certain item was clearly provided in the original literature, and a study with a total of scores > 7 was considered as a high-quality study.

### Statistical analysis

Two researchers (LFX and FX) repeatedly performed the processes of study screening and selection, data extraction, quality assessment of included studies, and data analyses independently. Any contradictoriness was resolved by discussion or an arbitrator (DJL) when consensus cannot be reached. Informed consent and ethics approvals were not required because all relevant data were extracted from published articles.

Meta-analysis was implemented to conduct the quantitative analysis and produce forest plots using Stata 12.0 Version (StataCorp, College Station, TX, USA). Evaluation parameters of interest involved the pooled RR as well as related 95% CIs was used to assess fracture risk between these two groups using random-effect models. Subgroup analyses were performed to explore the source of heterogeneity that was assessed using the *I*^2^ (statistically significant when *I*^2^ > 50%). Sensitivity analysis (conducted by omitting studies one by one) and publication bias (performed using Egger test) were implemented to evaluate the stability of results. A two-tailed *p* value < 0.05 indicated statistically significant.

## Results

### Study inclusion and exclusion

**Fig. 1:**
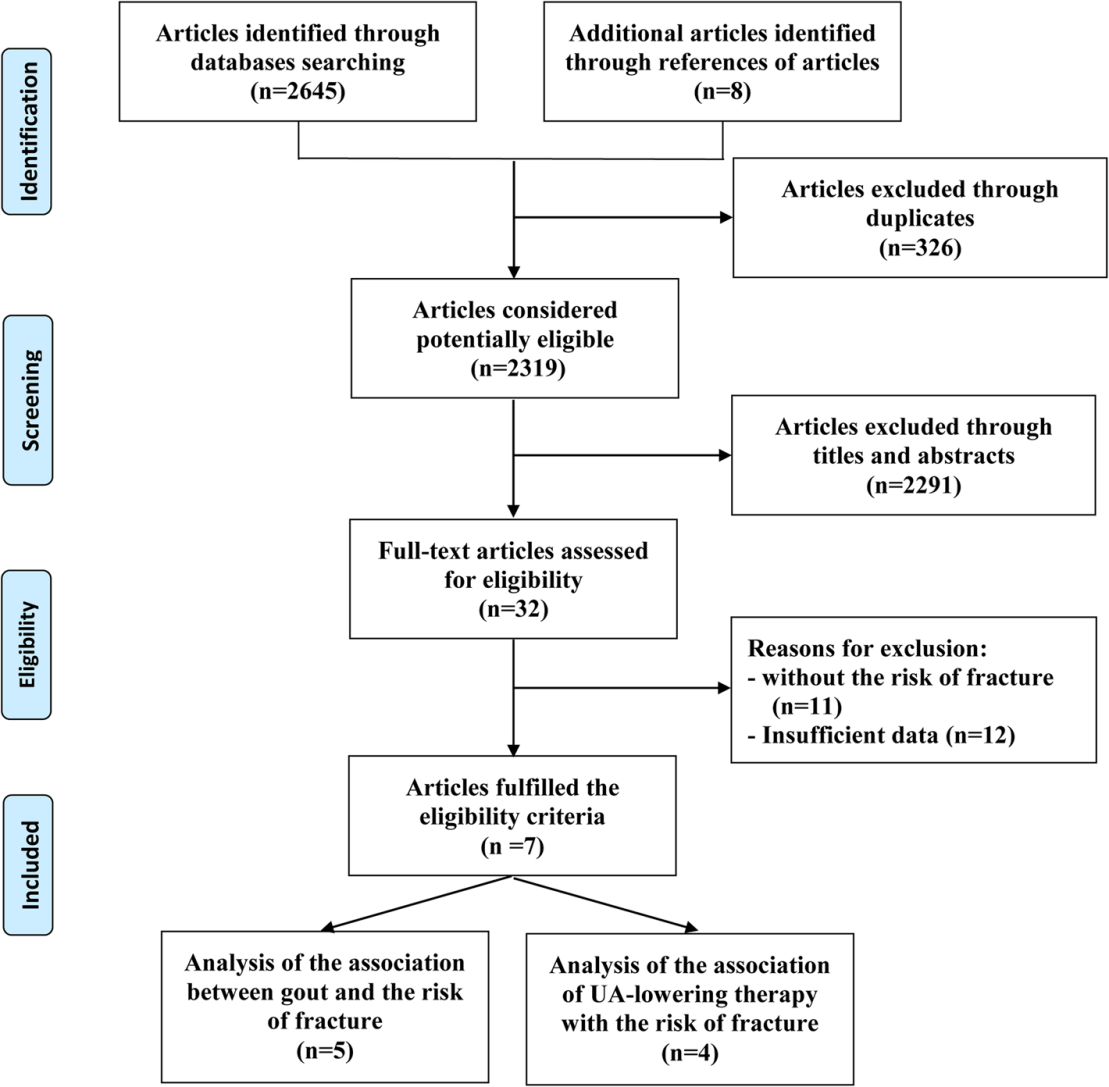
PRISMA flow diagram of literature research and selection process

A total of 2645 records were screened in the electronic databases searching process with another eight additional studies retrieved from screening reference lists of related articles. After 326 studies excluded by duplicates, 2291 excluded by reading titles and abstracts, and 32 studies were downloaded and carefully checked by reading full texts. After careful reading, five articles [1, 17, 23–25] involving eight datasets were considered to be qualified for the quantitative analysis of the association of gout with the risk of fractures, and four articles [1, 23, 26, 28] were considered to be qualified for the quantitative analysis of the association of urate-lowering with the risk of fractures. The inclusion processes and reasons for exclusion were depicted in Fig. 1.

### Search results and included participants

All included articles were published during 2016–2018 in English. Sample sizes of these studies ranged from 2674 to 292,808. With regard to the data source, all seven studies [1, 17, 23–26, 28] involved in the participants from the different data source. As for study type, five described retrospective cohort studies [1, 23–26] and two prospective cohort study [17, 28]. Kim et al. and Paik et al. [17, 25] presented data about the association between gout and fracture risk in two different cohorts based on the fracture localizations. Therefore, these data from the same articles were collected simultaneously and presented as two datasets based on sexuality. Eventually, this meta-analysis was established based on eight datasets. For the first analysis of gout with the risk of fractures, the baseline methodological and procedural characteristics of selected studies and demographic data of enrolled participants were listed in Tables 1 and 2. For the second analysis of the association of urate-lowering with the risk of fractures, Tables 3 and 4 show the baseline methodological and procedural characteristics of selected studies and demographic data of enrolled participants.

**Table 1:** Basic information of included studies

Author	Year	Region	Data source	Study design	Inclusion criteria	Inclusion interval	No. of patients		Outcome of interest (fracture site)
							Gout	Control group	
							group		
Tzeng, et al. [23]	2016	China	Longitudinal Health Insurance Database (LHID)	Retrospective cohort study	Gout patients were diagnosed, and the age was > 20 years old; Non-gout patients were randomly selected from LHID enrollees without any histories of gouts, and the selection frequency was at 1:2 ratios of age and sex.	January 01, 2001–December 31, 2009 The data were followed until December 31, 2011	43,647	87,294	Any fractures: hip, vertebral, wrist, humerus, other upper thigh, leg/knee, ankle/foot
Kim, et al. [25]	2017	USA	US commercial health plan	Retrospective cohort study	Gout patients were identified with ≥ 2 diagnosis codes and ≥ 1 dispensing for a gout-related drug. Non-gout patients, identified with ≥ 2 visits coded for any diagnosis and ≥ 1 dispensing for any prescription drugs, were free of gout diagnosis and received no gout-related drugs.	2004–2013 Patients aged ≥ 50 years who had ≥ 2 visits coded with the International Classification of Diseases	73,202	219,606	Hip, non-vertebral
Paik, et al. [17]	2017	USA	Nurses’ Health Study	Prospective cohort study	Participants were asked whether they had received a physician diagnosis of gout and the date of first occurrence on the questionnaires in 1982, 1984, 1986, and 1988. In 2002, participants were asked whether they had ever received a physician diagnosis of gout and the year of diagnosis.	1990–2004 for the wrist fracture analysis, 1990–2012 for the hip fracture analysis, patients age of 30–55 years	2225	101,574	Wrist, hip
Sultan, et al. [1]	2018	UK	Clinical Practice Research Datalink	Prospective cohort study	Gout patients were diagnosed based on a medical code assigned by the physician.	1990–2004 the data were followed until 2015	31,781	122,961	Non-vertebral, vertebral, wrist, hip, humerus
Wang, et al. [24]	2018	China	Shanghai metropolitan area	Retrospective cohort study	Gout patients were diagnosed by expert physicians based on to the International Classification of Diseases, 9th Revision, Clinical Modification (ICD 9-CM)	July 2014–July 2016	147	2527	Osteoporotic fracture

**Table 2:** Demographic characteristics of included participants

Author	Year	Female, *n* (%)		Mean age, years		Mean duration of follow-up, years		Comorbidities
		Gout group	Control group	Gout group	Control group	Gout group	Control group	
Tzeng, et al. [23]	2016	13,142 (30.1)	26,284 (30.1)	50.9 ± 16.0	50.9 ± 16.0	11	11	ALD, CAD, COPD, DM, ESRD, hypertension, osteoporosis, parkinson disease, stroke
Kim, et al. [25]	2017	13,176 (18.0)	39,529 (18.0)	60.0 ± 7.5	60.0 ± 7.5	2.0 ± 1.7	2.0 ± 1.8	Hypertension, stroke, cardiovascular disease, bone mineral density testing ordered, osteoporosis, dementia, chronic lung disease, chronic kidney disease, CAD, hyperlipidemia, obesity, DM, smoking, alcoholism, comorbidity score
Paik, et al. [17]	2017	2225 (100)	101,574 (100)	59.6 ± 6.5	56.0 ± 7.1	14 for the wrist fracture analysis; 22 for the hip fracture analysis	14 for the wrist fracture analysis; 22 the hip fracture analysis	Current smokers, hypertension, DM, osteoporosis, taking a thiazide diuretic
Sultan, et al. [1]	2018	8601 (17.1)	32,983 (16.8)	63.5 ± 12.5	63.1 ± 12.2	10.8 (6.7–13.4)*	10.8 (6.8–13.6)*	Smoking, alcohol consumption, history of falls, antihypertensive agents, antidiabetic agents, opioids, glucocorticoids, proton pump inhibitors, selective serotonin reuptake inhibitors, and bisphosphonates
Wang, et al. [24]	2018	75 (51.0)	1646 (65.1)	73.6 ± 12.0	75.6 ± 12.3	Not mentioned	Not mentioned	Rheumatic arthritis, CAD, hypertension, DM, physical activity, alcohol, smoking

*ALD* alcohol-related disorder, *CAD* coronary artery disease, *COPD* chronic obstructive pulmonary disease, *DM* diabetes mellitus, *ESRD* end-stage renal disease, *Median (IQR)

**Table 3:** Basic information of included studies

Author	Year	Region	Data source	Study Design	Inclusion criteria	Inclusion interval	No. of patients		Drugs
							Urate-lowering group	Control group	
Dennison, et al. [26]	2015	UK	Danish National Prescription Registry (DNPR)	Retrospective cohort study	All individuals who had at least one allopurinol prescription in the period from 1995 to 2011 in the Danish National Prescription Registry were identified. Patients were then further matched on quintiles of the propensity score to identify a more highly matched 1:1 control population.	1995–2011	86039	86039	Allopurinol
Tzeng, et al. [23]	2016	China	Longitudinal Health Insurance Database (LHID)	Retrospective cohort study	Gout patients were diagnosed, and the age was > 20 years old; Non-gout patients were randomly selected from LHID enrollees without any histories of gouts, and the selection frequency was at 1:2 ratios of age and sex.	January 01, 2001–December 31, 2009; the data were followed until December 12, 2011	6264 3747	728 3245	Allopurinol Benzbromarone
Basu, et al. [28]	2016	UK	Dundee social work database	Prospective cohort study	Analyses were confined to those aged 65 and over; those with a hip fracture sustained prior to the date of discharge from rehabilitation were excluded.	January 2001–December 2011	1115	16153	Allopurinol
Sultan, et al. [1]	2018	UK	Clinical Practice Research Datalink	Retrospective cohort study	Gout patients were diagnosed based on a medical code assigned by the physician and the landmark analysis was used to examine the effect of urate-lowering therapy on the risk of first fracture among patients with gout.	1990–2004; the data were followed until December 31, 2011	6750	24657	Not mentioned

**Table 4:** Demographic characteristics of included participants

Author	Year	Female, *n* (%)		Mean age, years (SD)		Mean duration of follow-up, years		Comorbidities
		Urate-lowering group	Control group	Urate-lowering group	Control group	Urate-lowering group	Control group	
Dennison, et al. [26]	2015	27,967 (33)	27,967 (33)	63 (15.1)	63 (15.1)	7	7	Allopurinol use, prior fracture, known comorbidity, drug history
Tzeng, et al. [23]	2016	Not mentioned	Not mentioned	Not mentioned	Not mentioned	11	11	Age, sex, ALD, CAD, COPD, DM, ESRD, hypertension, osteoporosis, Parkinson disease, stroke, and each drug
Basu, et al. [28]	2016	487 (43.7)	9684 (60.0)	73.2 (8.4)	73.4 (9.0)	12	12	Age, sex, creatinine, albumin, hemoglobin, diagnosis of diabetes mellitus, previous admission with ischemic heart disease, stroke, chronic heart failure, COPD, bisphosphonate prescription, vitamin D and calcium prescription, any exposure to allopurinol prior to the index date, and provision of a package of social care
Sultan, et al. [1]	2018	1925 (28.5)	6443 (26.1)	64.9 (12.0)	63.0 (12.5)	10.8	10.8	Age, alcohol consumption, smoking status, BMI, Charlson index, opioids, fall, glucocorticoids, nonsteroidal anti-inflammatory drugs, acetylsalicylic acid, proton pump inhibitors, antidiabetic and antihypertensive agents, and selective serotonin reuptake inhibitors

*ALD* alcohol-related disorder, *CAD* coronary artery disease, *COPD* chronic obstructive pulmonary disease, *DM* diabetes mellitus, *ESRD* end-stage renal disease

### Risk of bias

As for the risk of bias of included articles, six studies [1, 17, 23, 25, 26, 28] were rated as low risks in all nine items while the study conducted by Wang et al. [24] failed to describe the outcome assessment, resulting deduction of one score. All included studies were rated as high quality in the risk of bias assessment (Additional file 2: Table S1).

### Meta-analysis of any fracture

**Fig. 2:**
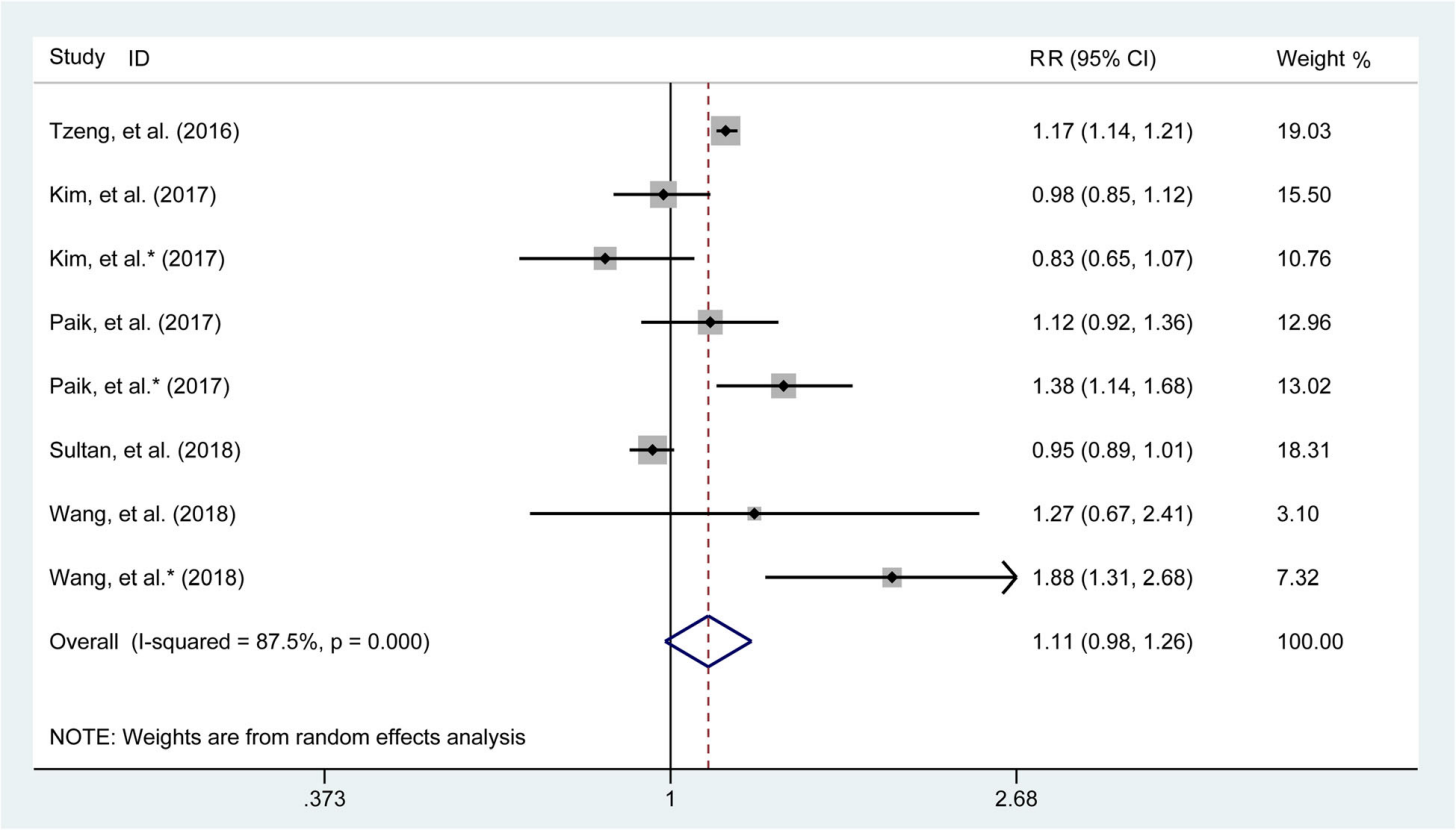
Forest plot involving the association of any fracture between gout patients and control participants. RR, relative risk; CI, confidence interval

The combined results as generated from five studies [1, 17, 23–25] involving 151,002 patients in the gout group and 533,962 participants in the control group recorded the frequency of any fracture demonstrated that gout was not associated with the risk of any fracture (pooled RR = 1.11, 95% CI 0.98–1.26, Fig. 2). Sensitivity analysis was conducted by omitting studies one by one (Additional file 2: Figure S1), subgroup analysis was implemented according to two factors including study design (retrospective or prospective) (Additional file 2: Figure S2), and the number of included patients (> or < 150,000 gout patients) (Additional file 2: Figure S3) revealed a consistent trend.

### Subgroup analysis based on sexuality

**Fig. 3:**
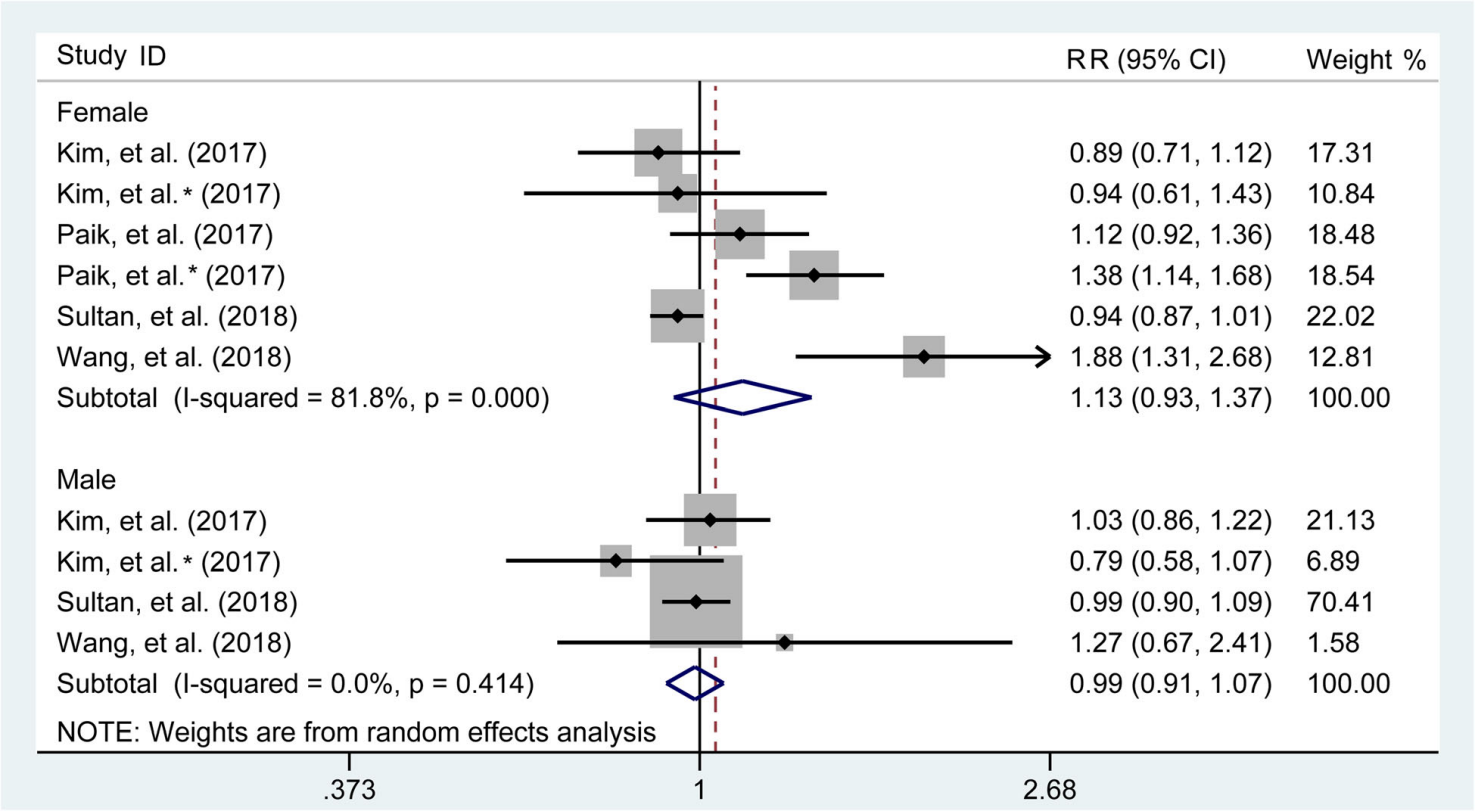
Subgroup analysis based on sexuality involving the association of any fracture between gout patients and control participants. RR, relative risk; CI, confidence interval

The combined results as generated from four [1, 17, 24, 25] and three studies [1, 24, 25] recording the number of fractures occurring on female and male, demonstrated that compared with the control group, the pooled RR for any fracture were 1.13 (95% CI 0.93–1.37) and 0.99 (95% CI 0.91–1.07) on female and male, indicated that sexuality had no effect on this association (Fig. 3).

### Subgroup analysis based on fracture sites

**Fig. 4:**
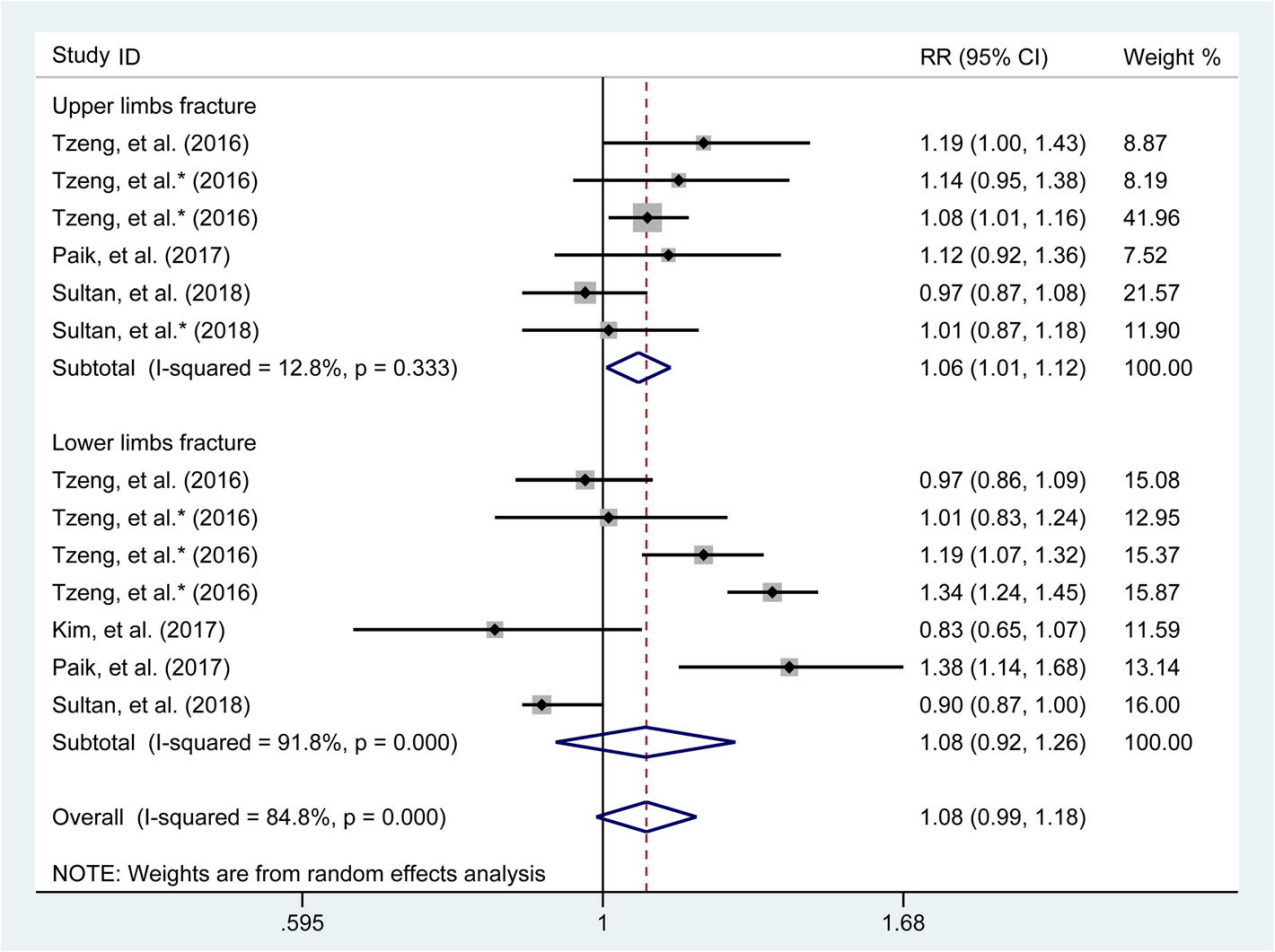
Forest plot involving the association of gout with fracture risk based occurring site (upper limb, lower limb). RR, relative risk; CI, confidence interval

**Fig. 5:**
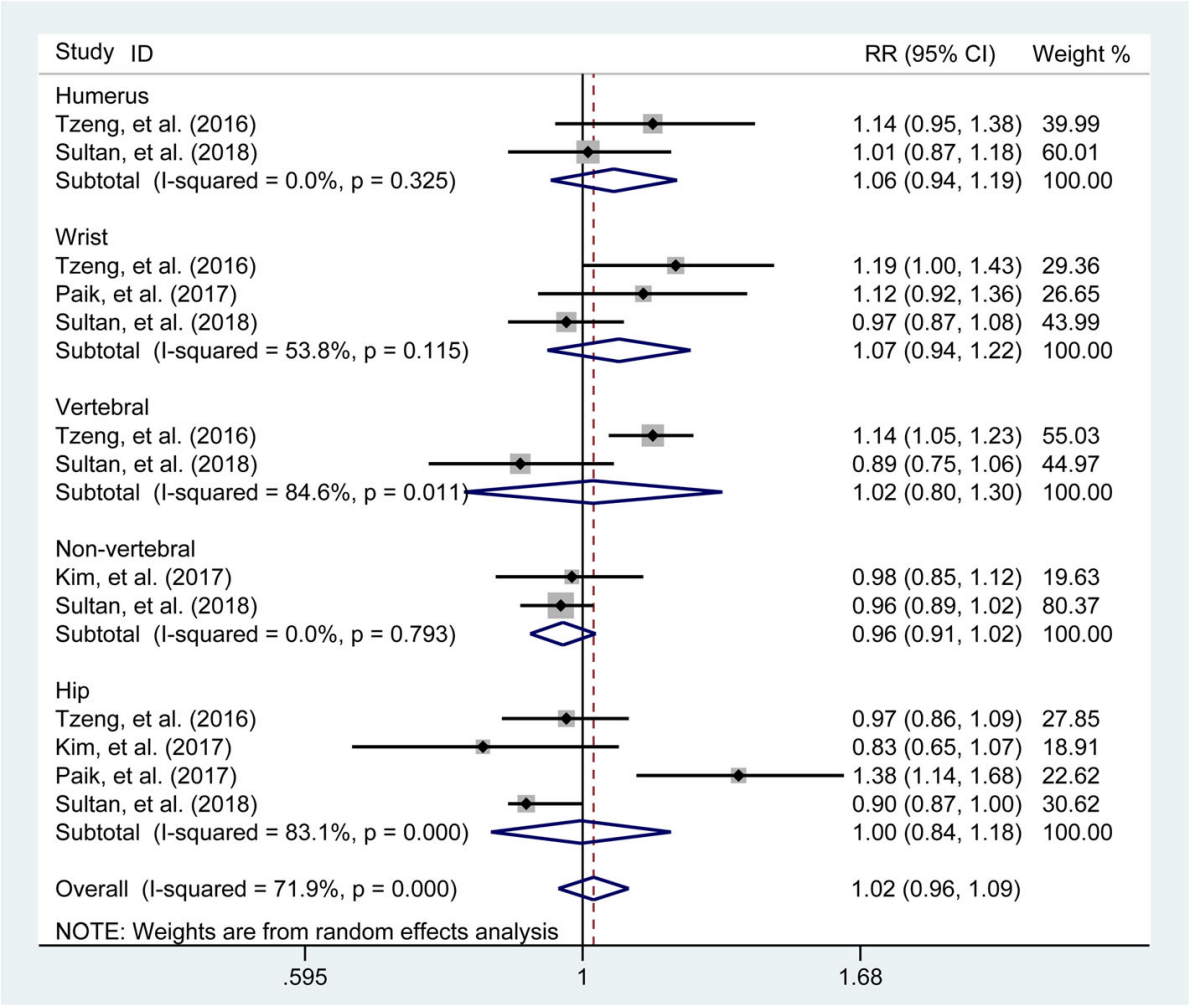
Forest plot involving the association of gout with the fracture risk based occurring site (humerus, wrist, vertebral, non-vertebral or hip). RR, relative risk; CI, confidence interval

The pooled results as generated from three [1, 17, 23] and four studies [1, 17, 23, 25] recording the number of fractures occurring at the upper limb and lower limb, respectively, showed that gout would not increase the risk of the lower limb fracture (pooled RR = 1.08, 95% CI 0.92–1.26) while showing a little increase in the risk of the upper limb fracture (pooled RR = 1.06, 95% CI 1.01–1.12) (Fig. 4). Further subgroup analyses were performed. There were two [1, 23], 3 [1, 17, 23], two [1, 23], two [1, 25], and four studies [1, 17, 23, 25] recording the number of fractures occurring at the humerus, wrist, vertebral and non-vertebral, and hip, respectively. The combined results showed that gout would not increase the risk of fractures (pooled RR = 1.06, 95% CI 0.94–1.19; RR = 1.07, 95% CI 0.94–1.22; RR = 0.96, 95% CI 0.91–1.02; RR = 1.00, 95% CI 0.84–1.18) when compared with those in the control group (Fig. 5).

### Association of urate-lowering therapy with the risk of fractures

**Fig. 6:**
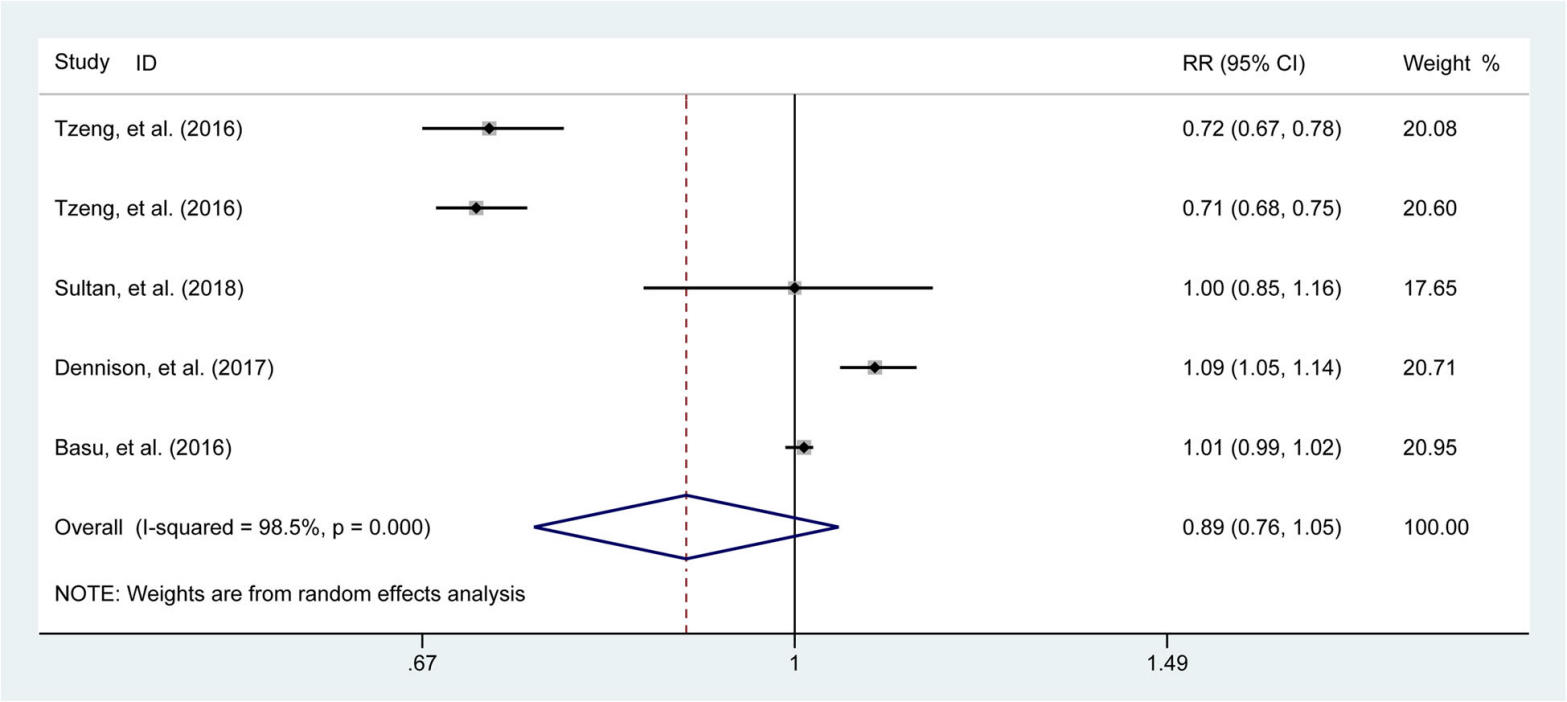
Forest plot involving the association of urate-lowering therapy with the risk for any fracture. RR, relative risk; CI, confidence interval

The combined results as generated from four studies [1, 23, 26, 28] recorded the use of urate-lowering drugs demonstrated that urate-lowering drugs prescribed early during disease had neither adverse nor beneficial effect on the long-term risk of fractures (pooled RR = 0.89, 95% CI 0.76–1.05, Fig. 6).

## Discussion

This meta-analysis aimed to address the fracture risk of patients with gout and demonstrated that gout was not associated with an increased risk of fractures for any site, such as the humerus, wrist, vertebral, and hip fracture. In addition, urate-lowering drugs prescribed early during the course of disease had neither adverse nor beneficial effect on the long-term risk of fractures.

As a typical feature of gout, high UA levels were demonstrated an inconsistent relationship with BMD and fracture, because the diverse properties of UA depend on the specific conditions. The protective effects of UA on BMD are mainly because of the antioxidant function or via vitamin D/parathyroid hormone pathway [29–31]. The pro-oxidant properties are dominant when UA exists at supersaturated concentrations, such as in gout, and results in oxidative stress [32, 33]. Numerous cross-sectional analyses demonstrated the positive or inverse association between UA and BMD [18–21]. The more recent one based on the National Health and Nutrition Examination Survey (NHANES) reported the neutral results which were also supported by animal data [22]; however, a prospective Rotterdam Study revealed that a higher UA level led to a higher BMD [34]. In terms of the association between UA and fracture risk, the results also remained inconsistent, which may be partly related to the age and sex distribution in different studies. A prospective Osteoporotic Fractures in Men (MrOS) study recruiting 5994 men aged 65 years or old showed a higher UA level had a negative impact on the risk of non-spine fracture, but not applied in hip fracture [35]. A more recent prospective study included 1963 men and 2729 women and found a significant positive association between high UA level and an increased risk of hip fractures, but only in men [36].

Hyperuricemia is a necessary but not sufficient predisposing factor for gout [23], which is also characterized by uric acid crystals and inflammatory. Importantly, the uric acid crystals can activate the NOD-like receptor protein-3 (NLRP3) [37, 38], which act as a diver of pro-inflammatory cytokines. Additionally, interleukin-1 beta (IL-1β), a strong stimulator of bone resorption, can be produced in the inflammatory cascades during gout [39–41]. These effects further promote osteoclastogenesis and osteoclast differentiation and may be related to the changes in BMD, thereby potentially increasing fracture risk [42–46]. Therefore, the complex pathophysiology made the relationship between gout and fracture varied.

Our research based on the analysis of seven studies including 151,002 gout patients and 533,962 participants in the control group revealed that there was no significant association between gout and fracture risk. Three of these articles demonstrated different conclusions from ours. Although Tzeng et al. [23] found that gout increased the overall fracture risks, the absent detailed information regarding important lifestyle-related factors (body mass index, smoking status, and alcohol habit) in the National Health Insurance research database (NHIRD) may confound their conclusions. The large prospective cohort study performed by Paik and colleagues showed that a history of gout modestly increased the risk of hip fracture in women but was not associated with wrist fracture [17]. However, the population in this study was female, so their findings should be carefully generalizable to men, while our subgroup analysis based on gender did not show significant a significant increase in the risk of fractures. Wang et al. [24] demonstrated that, in Chinese adults, gout significantly increased the risk of osteoporotic fractures in women. Because of its cross-sectional design, some biases were inherent, and the causal relationship between gout, fractures, and osteoporosis cannot be established; therefore, the results required cautious interpretation. Notably, a subgroup analysis based on the site of fractures was conducted in our research; the results showed that gout increased the risk of upper limb fractures, but a more detailed location analysis (humerus, wrist, and etc.) made the relationship disappear. Therefore, more prospective studies focusing on different factors should be performed to identify the association between gout and high fracture incidence under specific conditions.

We found that gout-related medication had a neutral effect on the risk of fractures, although previous literature on this topic was conflicting. Dennison et al. [26] reported that 9% excess risk of osteoporotic fractures was associated with gouty arthritis requiring allopurinol; however, the significant differences of characteristics between the exposed group and nonusers, and the control group involving non-gout participants may affect their observation. The opposite conclusion was raised by Tzeng et al. [23], in which urate-lowering therapy decreased a 28% risk of fractures among gout patients, while this study ignored the exclusion of the patients in the exposed group who underwent other events before the first prescription of urate-lowering therapy. A cohort study conducted by Sultan et al. demonstrated that urate-lowering therapy had a neutral effect on the long-term risk of fractures [1]. Although the use of 1- and 3-year landmarks to some extent limited the generalization of the population, the nationally representative data recording enables us to understand better the causal relationship between therapy and fractures with minimum bias.

The strength of this study compared with previous investigations lies in the large quantity of included data, reliable statistical methods, and comprehensive evaluation indexes. This meta-analysis included eight datasets by exhaustive database searching and reference screening, among which there were seven datasets from national or regional register databases. The large participant base made the results of this study well represented. Moreover, outcomes of interests were compared using a pairwise meta-analysis method and analyzed from multi-dimension to fully elucidate the association between gout and the risk of fractures and discuss the underlying mechanism. Findings of this meta-analysis provide the currently most comprehensive evidence about this issue, which will help gout patients and their clinicians to determine whether or not to make an appropriate therapeutic regimen to propose the fracture. Hence, the conclusions based on these articles were not fairly reliable.

Nevertheless, this investigation was not without limitations. First, we only demonstrated an overall effect of gout on fracture but were not able to analyze the influence of some clinical and demographical factors (e.g., age and comorbidities). Second, there were only two prospective studies, and no randomized controlled trials were included. However, given the properties of gout and source of data, large scale RCT is currently inaccessible. Third, several studies did not present all characteristics of participants, and these missing data affect and weaken the strength of conclusions.

## Conclusion

This meta-analysis confirmed that gout was not associated with an increased risk of fractures. Urate-lowering drugs prescribed early during the course of disease had neither adverse nor beneficial effect on the long-term risk of fractures.

## Additional files

Additional file 1: PRISMA checklist. The checklist of the Preferred Reporting Items for Systematic Reviews and Meta-Analyses (PRISMA). (DOC 64 kb)

Additional file 2: Supplementary figures 1–3 and table 1. (DOC 2252 kb)

## Data Availability

All data generated or analyzed during this study are included in this published article.

## Abbreviations

BMD: Bone mineral density
CIs: Confidence intervals
IL-1β: Interleukin-1 beta
MrOS: Osteoporotic Fractures in Men
NHANES: National Health and Nutrition Examination Survey
NHIRD: National Health Insurance Research Database
NLRP3: NOD-like receptor protein-3
NOS: Newcastle-Ottawa Scale
PRISMA: Preferred Reporting Items for Systematic Reviews and Meta-Analyses
RR: Risk ratios
UA: Uric acid
