# Supplementary material for: Gout is not associated with the risk of fracture: a meta-analysis
Source: J Orthop Surg Res. 2019 Aug 27;14:272. doi: 10.1186/s13018-019-1317-4 (PMC6712626; doi:10.1186/s13018-019-1317-4)
Supplement: Supplementary file 2 — Supplementary figures 1–3 and table 1. (DOC 2252 kb) [file 13018_2019_1317_MOESM2_ESM.doc]

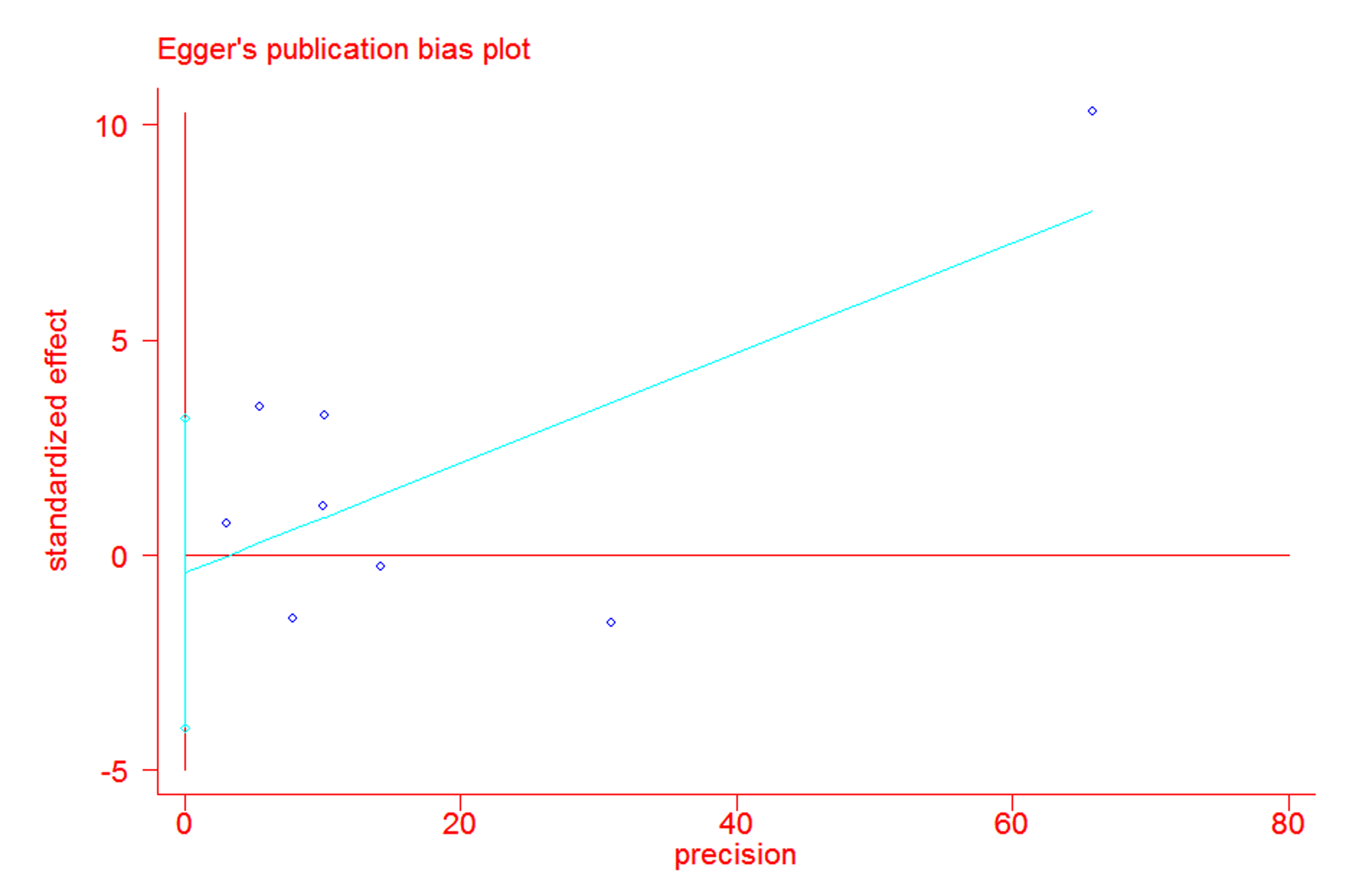


**Supplementary Figure 1. Publication bias plot of Egger’s test (*p* = 0.789).**


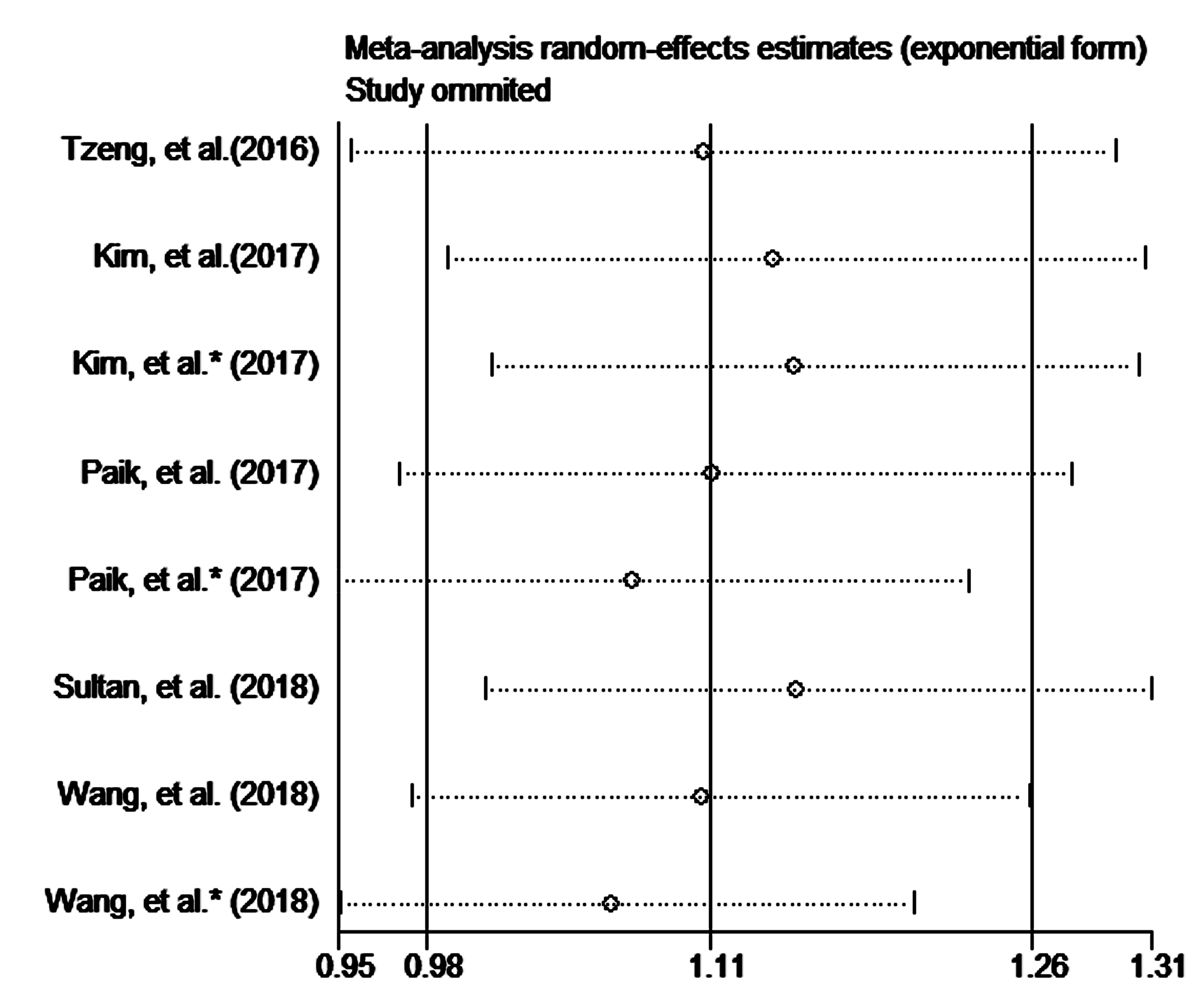


**Supplementary Figure 2. Sensitivity analysis about any fracture by omitting studies one by one.**


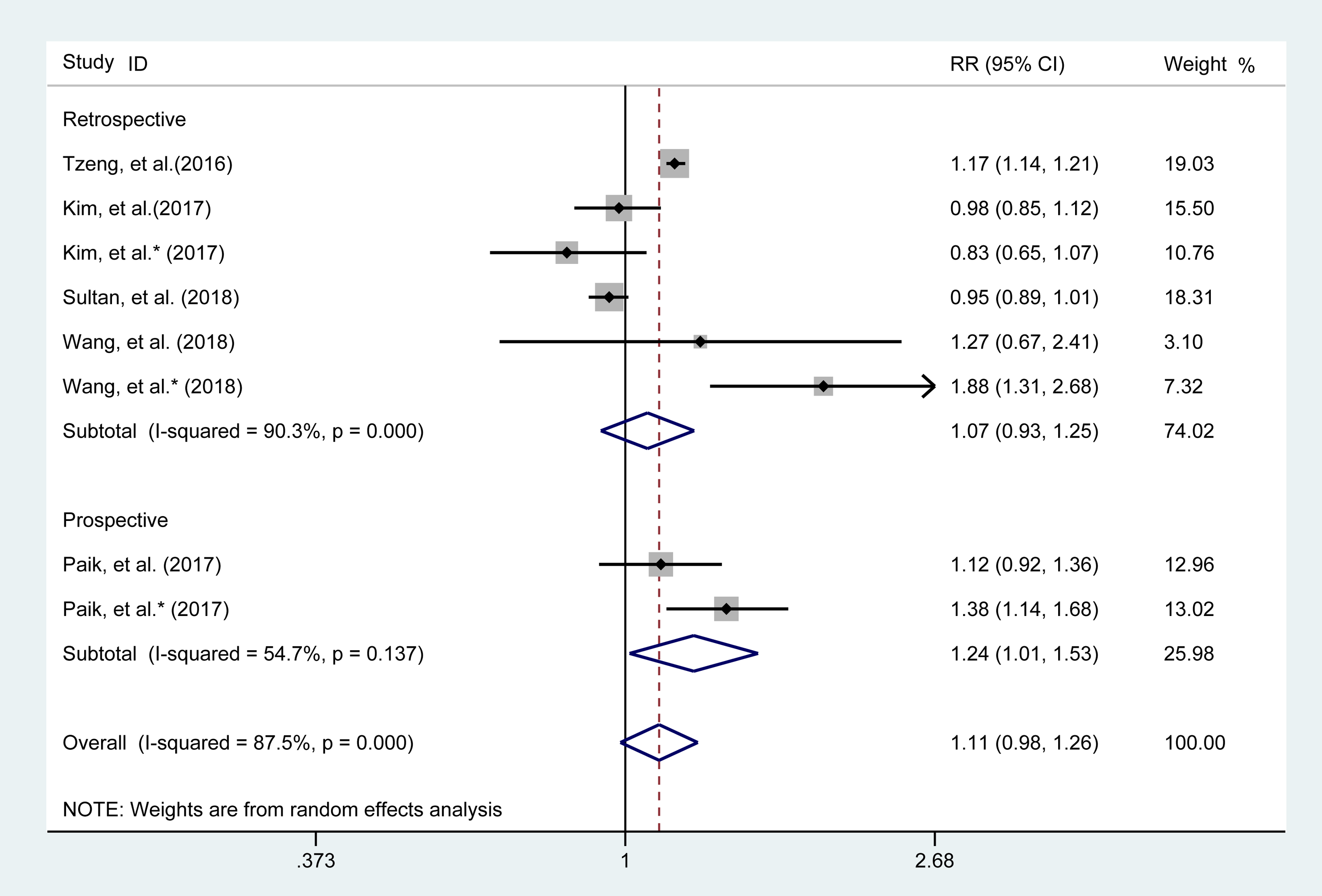
**Supplementary Figure 3. Subgroup analysis of any fracture based on the study design.**

The diamond in the right of the central vertical line represents higher prevalence of fracture in the gout group in comparison with the control group. RR, risk ratio; CI, confidence interval.


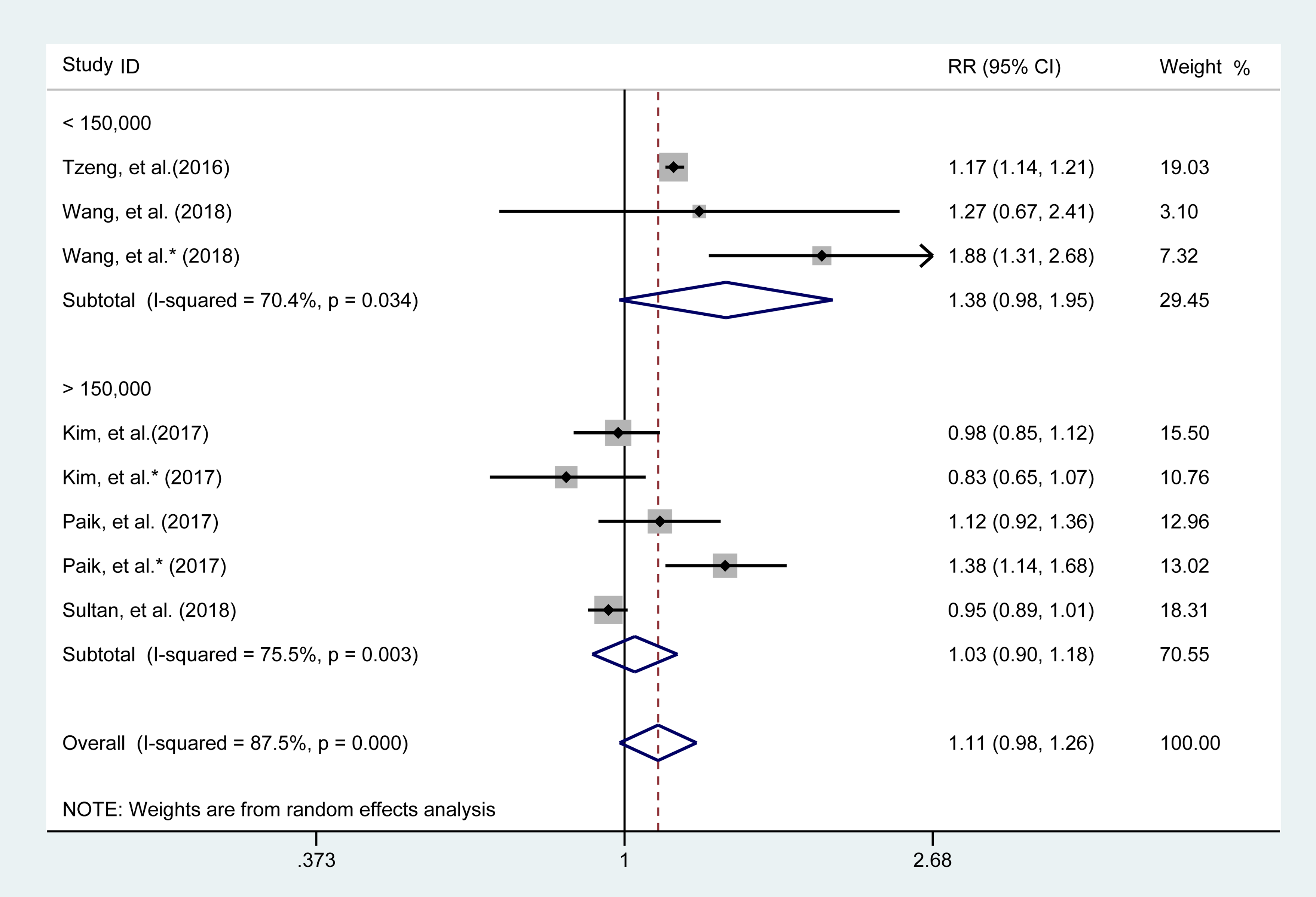


**Supplementary Figure 4. Subgroup analysis about fracture risk based on the sizes of included subjects.**

The diamond in the right of the central vertical line represents higher prevalence of any fracture in the gout group in comparison with the control group. RR, risk ratio; CI, confidence interval.

**Supplementary Table 1. Quality assessment of included studies.**

| Study, years | Items | | | NOS | Study Design |
| --- | --- | --- | --- | --- | --- |
|  | Selection | Comparability | Outcome/Exposure |  |  |
| Tzeng, et al. 2016 | **** | ** | *** | 9 | cohort study |
| Kim, et al. 2017 | **** | ** | *** | 9 | cohort study |
| Paik, et al. 2017 | **** | ** | *** | 9 | cohort study |
| Sultan, et al, 2018 | **** | ** | *** | 9 | cohort study |
| Wang, et al, 2018 | **** | ** | ** | 8 | cohort study |
| Dennison, et al. 2015 | **** | ** | *** | 9 | cohort study |
| Basu, et al. 2016 | **** | ** | *** | 9 | cohort study |

NOS, Newcastle-Ottawa scale.
